# The careful use of opioids and alternatives for pain management

**DOI:** 10.1016/j.ebiom.2025.105851

**Published:** 2025-07-09

**Authors:** eBioMedicine

In the USA in 2023, the National Health Interview Survey found that 24.3% of adults experienced chronic pain in the past 3 months, and 8.5% experienced chronic pain that limited their daily work and activities. Women were also found to have higher pain levels, and pain levels increased with age. Whether acute, chronic, neuropathic, or inflammatory, pain is a major source of loss of quality of life and has a high socioeconomic cost. Due to their efficacy in controlling pain, opioids are often prescribed after other analgesics, such as ibuprofen, have proven insufficient.

However, opioids are highly addictive and are likewise associated with harmful health and societal problems. Most data describing opioid use disorder (OUD) are from the USA, where widespread opioid prescriptions might have contributed to the opioid crisis and thousands of preventable deaths. Although the Centers for Disease Control and Prevention reported a slight decrease in deaths involving synthetic opioids other than methadone—from 73 838 in 2022 to 72 776 in 2023—the overall costs of opioid use remain very high, underscoring the need to carefully weigh their benefits against their risks and the burden of pain.

According to National Institute for Health and Care Excellence guidelines, there is little evidence that opioids are effective for primary pain in the long term. Although usually intended for short-term symptom management while the underlying cause is being identified or treated, chronic pain that is difficult to otherwise treat often results in the long-term monitored use of opioids.

Mechanisms involving both peripheral nerve activation, due to tissue injury or inflammation, and CNS processing produce painful sensations, which are an alert to a health concern. Opioids bind to receptors in the CNS, which contributes to analgesia while also producing euphoria. Unfortunately, a costly side-effect of this is their addictive properties, because of their ability to reinforce use mediated by increased dopamine release in reward pathways. Tolerance and dependence develop as a result of neuroadaptive changes, such as receptor desensitisation and downregulation, in response to repeated opioid use. Respiratory depression occurs when opioids activate mu opioid receptors in brainstem respiratory centres, diminishing the brain's responsiveness to carbon dioxide concentrations and impairing breathing.

Non-addictive pharmacological alternatives to opioids for pain management is a growing area of research. Published in *Cell* in May 2025, Guo and colleagues presented the success of SBI-810, an arrestin-biased allosteric modulator of neurotensin receptor 1, in preclinical models to alleviate acute and chronic pain without addiction. SBI-810 was administered to mice for up to six days, and researchers found that it selectively suppressed neuronal excitability and central sensitisation by inhibiting NMDA receptor activity, ERK signalling, and sodium channel expression in nociceptive neurons, without impairing motor or cognitive functions. The compound showed more efficacy than gabapentin at reducing neuropathic pain from spared nerve injury. When used in conjunction with opioids, opioids were more effective at lower doses. Researchers also found less drug-seeking behaviour and withdrawal symptoms in the mice, while maintaining stable analgesia without tolerance or sensitisation.

However, the intervention period, although typical for these studies and sufficient for the assessment of tolerance, might not fully recapitulate the extended duration of human long-term use. Furthermore, although it is promising in the animal models, differences in pain receptors and pathways, pharmacokinetics, and pharmacodynamics, as well as behavioural and cognitive differences, challenge translation of preclinical analgesic studies. Human neurons treated by SBI-810 had reduced action potential firing, suggesting similar effects in humans, but human trials are needed.

A substance use disorder is defined as occurring when an individual loses control over their substance use. This can develop as a result of opioid use, particularly when used for extended periods, due to the consequential development of tolerance and addiction. Assessing the effects of addiction in non-human primates, a study published in *eBioMedicine* showed that three years of oxycodone use was associated with lower grey matter volume in specific brain regions implicated in executive functioning, sensory processing, motor control, and reward-related behaviours. Additionally, researchers found increased expression of neurodegenerative biomarkers.

Until successful opioid alternatives are widely available, polygenic risk scores (PRS) are a valuable tool for enabling personalised medicine to assess risk associated with opioid prescriptions. However, they do not provide the full picture of risk. For instance, in a 2025 PRS study of 1958 adults of European ancestry, although the PRS for OUD were positively associated with opioid dependence, household income and education had the strongest correlation. PRS are not yet routinely used in clinical practice to guide opioid prescribing, but when they are, they should be considered in the context of other clinical assessments and monitoring to avoid false negatives and false positives.

Environmental stressors are central in influencing the risk of addiction. For example, a retrospective cohort study of 4,876,209 US women in *The Lancet Regional Health Americas* showed that military service women and civilian dependents had a significantly increased risk of a sustained prescription of opioids during a period of increased operational intensity. This is defined as a period during which military operations and combat are more frequent or widespread, and researchers believe the findings reflect emotional distress, insufficient support, and military sexual trauma.

Opioid use does not exist in a vacuum, and comorbidities associated with OUD can complicate current understanding of the risk of addiction. A review published in *eBioMedicine* found evidence of an underlying genetic liability that predisposes individuals to both substance use disorders and chronic pain, as well as conditions such as depression and cardiometabolic disease. In a 2022 study in *Neurophsychopharmacology* examining 114 healthy adults of European ancestry, researchers found a higher PRS for major depressive disorder was associated with increased μ-receptor availability in the amygdala and ventral pallidum. These brain regions are central components of the limbic and reward circuits. Both major depressive disorder and OUD genetic risks influenced how the brain's opioid system responded to stress, with more pronounced effects in women.

However, in *PLOS One*, a study of 1103 patients of African-American ancestry demonstrated that the PRS for lifetime cannabis use and heavier drinking were both associated with an increased risk of opioid misuse in men. A genome-wide association study published in *Nature Mental Health* in 2023 derived a general addiction risk factor PRS through multivariate analysis that influenced susceptibility to addiction across various substances, including opioids. Across ancestries, *PDE4B* was important, which has a role in dopamine regulation. One opioid-specific locus was associated with opioid addiction, rs1799971 in *OPRM1*, the opiate receptor μ1 gene.

Emerging alternative strategies, both pharmacological and non-pharmacological, continue to offer renewed hope for more effective and safer pain management. A meta-analysis in *The Lancet Neurology* of 284 pharmacological and 29 neuromodulation studies showed a basis for the recommendation of the use of tricyclic antidepressants, α2δ-ligands, and serotonin and norepinephrine reuptake inhibitors as first-line treatments for neuropathic pain.

Psychological interventions traditionally include the addressing of emotions, beliefs, and behaviours. These often fall under the banner of cognitive behavioural therapy. For example, cognitive therapy, mindfulness-based stress reduction, and behaviour therapy performed better than treatment as usual in a study of 521 patients with lower back pain, emphasising that techniques rooted in evidence-based psychosocial treatments have high efficacy in pain management. This is thought to be linked to these treatments’ ability to improve physical function, sleep, and mood. Transcutaneous electric nerve stimulation is another avenue of research, showing some evidence of benefit for acute and chronic pain management, but there is inadequate standardisation of transcutaneous electric nerve simulation protocols. Related to this, memory reconsolidation updating procedure timed within hours of methadone dosing enhances treatment outcomes in opioid use disorder, reducing relapse risk. This timing aligns with the reconsolidation window, during which drug-related memories become more susceptible to modification, thereby weakening the associations that contribute to craving and relapse.

It is crucial that the underlying health concerns that are causing pain are addressed to avoid reliance on long-term pharmacological interventions such as opioids, which carry substantial risks. A comprehensive approach to tackle pain is also crucial, incorporating personalised therapies and psychological and lifestyle factors. At *eBioMedicine*, we encourage submissions that elucidate pain and addiction mechanisms, explore pharmacological and non-pharmacological alternatives to opioids, and promote careful or personalised prescribing—aiming to transform the future of pain management.

